# Video Self-Modeling Is an Effective Intervention for an Adult with Autism

**DOI:** 10.1155/2014/425897

**Published:** 2014-09-03

**Authors:** Genevieve Hin Ha Tsui, M. D. Rutherford

**Affiliations:** Department of Psychology, Neuroscience and Behaviour, McMaster University, Hamilton, ON, Canada L8S 4K1

## Abstract

With the increases in size and strength that come with adulthood, challenging behaviours among those with autism spectrum disorders (ASD) can become critical. Few studies have explored behavioural interventions in adults with ASD, though recent studies have shown video self-modeling (VSM) to be effective in children with ASD. VSM involves an individual watching videos of himself demonstrating prosocial behaviours, while those behaviours are pointed out and encouraged. In the current study, VSM was used to encourage prosocial behaviours and to reduce problematic behaviour displayed by an adult with ASD. Results reveal a decrease in the tendency to invade others' personal space and make inappropriate loud noises. VSM may be an effective intervention and improve the lives of adults with ASD.

## 1. Introduction

Autism spectrum disorders (ASD) are a spectrum of disorders including Rett's syndrome, childhood disintegrative disorder, Asperger's syndrome, and pervasive developmental disorder-not otherwise specified (PDD-NOS), together characterized by deficits in two areas: (1) social communication and interactions and (2) restricted patterns of behaviours and interests [1]. ASD has a prevalence of 13/10,000 [2]. Since ASD is a pervasive disorder with great variability of symptoms, a broad range of treatment and intervention is employed in treating ASD [3].

Increasing attention has been given to identifying and providing targeted interventions to address the challenging behaviours that are common among those with ASD. Some of these behaviours include aggression, destructive behaviour, and self-injurious behaviour. These behaviours can lead to self-harm and the harm of others, employment and housing challenges, and a wide range of other negative consequences.

ASD is a lifelong disorder, but more research has been conducted on treatment in childhood than on adult treatments and interventions [4]. Research into treatment of adult behaviour is important because, with increased physical size and strength in adolescents and adults, maintaining a safe environment becomes more difficult [4]. Socially inappropriate behaviours can lead to problems with independent living and adversely impacting social interactions, access to community-based services, and employment [5]. Such behaviours can also interfere with learning, the acquisition of professional skills, and the acquisition of prosocial and adaptive behaviours [4].

### 1.1. Video Modeling

One approach that may be an effective intervention for adults with ASD is a video intervention, designed to improve skills or reduce problematic behaviours in individual with ASD by modeling appropriate behaviours. This intervention involves the participant watching videos of a model performing a target behaviour or a skill that is being taught [6]. Since symptoms of ASD vary considerably across those with ASD, this intervention is individualized according to the needs of each participant [6]. Video modeling has been shown to be an effective intervention in children with ASD [6].

Charlop-Christy and colleagues [7] compared real-time modeling, that is, having the observer watching live models performing the target behaviour, and video modeling in teaching developmental skills, such as oral comprehension, spontaneous greetings, independent play, and social play, to children with ASD. They found that video modeling resulted in faster acquisition of tasks than real-time modeling. Video modeling but not real-time modeling also led to generalization of these tasks across different settings, stimuli, and people [7].

### 1.2. Video Self-Modeling

The most effective video interventions involve models who are matched to the participant in terms of age, gender, and race, as who perform only slightly better than the observer [8]. To create an even better match between model and participant, self-modeling can be used. Here, a video is created using the participant himself or herself as the model, maybe even more effective than peer modeling in teaching the participant new skills or positive behaviours and reducing the undesired behaviours [8].

Video self-modeling (VSM) in which the video displays the individual who is to be trained became technically possible and was first described in the early 1970s [9]. In the video self-modeling intervention, a video of the observer performing only the targeted skills or behaviours would be created. One method of creating the video required the observer to role-play or imitate the target behaviour while he or she was being videotaped [6]. This method was particularly effective for social skills which often involved imitations [10]. Another method was to record the observer's spontaneous behaviour over time then editing the video to include only the desired behaviours [6]. In either case, the individual would then watch the video on a regular training schedule and would be given opportunities to participate in the situations depicted in the video [6].

Several studies examined VSM effects on social communication skills in students with ASD. One of these studies was by Buggey et al. (1999), in which VSM was used to train students with autism to responding appropriately to questions being asked. They found participants' mean appropriate responses approximately doubled after the intervention [11]. Wert and Neisworth (2003) examined the effect of VSM on spontaneous requesting of children with autism, encouraging them to ask for an object or action. Every participant in this study demonstrated a significant increase in spontaneous requesting following the VSM intervention [12].

More recently, Buggey (2005) investigated whether VSM was effective in modifying behaviours such as language production, social initiations, tantrums, and aggressive pushing, of children with ASD [8]. Positive outcomes were found for all behaviours and with all participants, supporting previous evidence of the success of VSM. Furthermore, although the intervention took place in school classrooms, changes in behaviour were generalized to other settings within the school. These behaviours were also maintained after the intervention, and the target behaviours were produced shortly after the intervention was introduced.

Finally, one recent study found evidence that VSM was an effective intervention in teaching job skills. Three adults with intellectual disabilities (one who had ASD) were shown videos that demonstrated a specific chain of job tasks. VSM was found to facilitate task acquisition [13].

### 1.3. Current Study

Previous studies demonstrating the effective use of VSM in ASD populations focused on children rather than on adults. The current study attempted to gain some insight into the effectiveness of VSM in shaping prosocial behaviours and reducing problem behaviours of an adult with ASD. The participant in this study (JD) was chosen because he showed severe yet specific socially inappropriate behaviours. The problem behaviours of interest were the participants' tendencies to make unprovoked loud noises and to invade the other's personal space. By introducing the VSM intervention to this participant, it was hoped that there would be a reduction in the frequency of his problem behaviours.

## 2. Method

### 2.1. Participant

The participant, JD, was a 30-year-old Caucasian male who had been diagnosed with classic autism at the age of 2. His diagnosis of ASD was affirmed in his late 20's by a clinician before entering his current treatment program and was confirmed by an ADOS for inclusion in this study using the ADOS-2 [14]. At the time the study started, he was already participating in programming at Salvation Army Lawson Ministries of Hamilton, Ontario. This program was a daily out-patient intervention that involved intensive social skills training as well as living skills training (e.g., laundry, cooking, and fitness). He was selected for inclusion because, prior to the study, he exhibited several unwanted behaviours: making spontaneous loud noises and invading others' personal space by attempting physical contact, including touching and kissing.

### 2.2. Apparatus

Test videos were taken with a Sony Handycam HDR-CX190 video camera on a tripod. The intervention video was taken with the same video camera and was edited using the iMovie program of an Apple MacBook Pro laptop.

### 2.3. Experimental Design and Procedure

Effect of the intervention was primarily tested by comparing the participant's behaviour before and after the 4-week intervention (described below), as recorded and coded from video by two naïve coders. All videos were recorded at the Salvation Army Lawson Ministries Hamilton. This is a nonresidential treatment facility designed specifically for older adolescents and adults with ASD to receive support around social skills and living skills. JD viewed the intervention video in a well-lit room at Lawson Ministries building. Video coding took place in a well-lit, quiet laboratory room with no distractions.

### 2.4. Baseline Videos

Six baseline videos, ranging in length from 1 minute to 5 minutes, were recorded prior to the intervention. These videos were created following the completion of a survey of those staff members who worked with JD. The survey consisted of five questions about the problematic behaviours and the situations most likely to elicit the behaviours (see the appendix). Note that the questions on the questionnaire were open ended: it did not include a list of possible behaviours or definitions or examples of problematic behaviours. The staff were free to nominate any behaviour they thought to be problematic. Six staff members of Lawson Ministries of Hamilton completed the survey, and these six staff members were chosen because they worked with JD at least several times a week. The behaviours identified in this survey became the focus of the study.

Videos were recorded during the times when JD's problem behaviours were most likely to occur, according to the survey. To record these videos, a video camera was set up on a tripod and positioned inconspicuously in the corner of a room, prior to the arrival of JD. Once the video camera was set up, JD was brought into the room and seated at the table where he could be recorded. Although he had previously consented to participation in this study, the videotape was recorded without alerting him that he was being recorded at that moment. During the recordings, JD was asked to work on a task that required concentration, such as a puzzle or math problems. While he was concentrating on his task, a staff member was instructed to enter the room and greet him. Following a short interaction with JD, the staff member left the room, leaving JD to work on the task alone. Each video started before the arrival of the staff member and ended shortly after the staff member left. No edits were made to these videos.

### 2.5. Creating the Intervention Videos

In order to create the intervention video, JD was directed to imitate several prosocial behaviours, initiating a proper handshake with eye contact, greeting politely by saying “hi,” and keeping a distance away when being greeted. Each behaviour was first named and then modelled, and then JD was given the opportunity to demonstrate the behaviour. His attempts were praised (regardless of accuracy). If the attempt was inadequate, then corrections were described as specifically as possible, modelled again, and JD was given another chance to display the behaviour. Once performed correctly, these behaviours were videotaped. The video was edited to only include the desired behaviours. In particular, undesired behaviours, such as attempts to invade others' personal space or making loud noises, were eliminated. The video paused following the completing of each target behaviour, and a narrator named and commented on the appropriate behaviour that JD had just completed. The intervention video was approximately 3 minutes in length.

### 2.6. Intervention

JD viewed the intervention video 3 times a week, for 4 consecutive weeks. On those days on which he viewed the video, he viewed it upon his arrival to Lawson Ministries, before he began his regular scheduled day program. If JD looked away while the video was playing, he was prompted to direct his attention back to the video. JD was praised for paying attention after watching the video.

After each viewing, a practice period of approximately 10–15 minutes followed. In this practice period, JD practiced greeting staff members in a polite manner when he encountered them. He was prompted to make a proper handshake and make eye contact, reply politely by saying “hi,” and respect others' personal space. This allowed JD to practice the behaviours that were portrayed in the video and served as a check that he was paying attention to the video. Verbal praise was given immediately when JD successfully performed the appropriate behaviour.

### 2.7. Postintervention

A set of 6 test videos, ranging in length from 1 minute to 5 minutes, was recorded following the 4-week intervention period. These videos were designed to mirror the situations staged in the baseline videos. They were recorded using the same settings, situations, and activities as the baseline videos. They were recorded at the same time during the day as the baseline videos. No edits were made to these videos.

### 2.8. Follow-Up Interview

Following the intervention, an interview was conducted with the six staff members who took the survey before the intervention began. Data obtained from these interviews were compared to the data obtained from the surveys that were conducted before the intervention. Interviews with staff were conducted in a well-lit room at Lawson Ministries building.

## 3. Results

Evaluation of JD's behaviour was completed by 2 video coders, who each viewed every baseline and test video and who coded the videos independently. Coders were also blind to the type of video (baseline or postintervention) they were coding. The orders of the type of videos presented to the coders were counterbalanced.

Prior to coding the videos, each coder received a 1-hour training session in order to teach them the coding procedures. They were instructed to observe and record the duration of specific behaviours displayed by JD onto a coding sheet. Each behaviour was defined, and any questions the coder had were answered. Coders were asked to record each example of the behaviour and its starting and ending time. Instructions to coders are found in the appendix.

While coding the video, coders worked from a list of negative and target behaviours that were of interest. Coders viewed each video twice, one time focusing only on the appropriate behaviours and one time focusing on the problematic/inappropriate behaviours. They recorded the behaviour identified and recorded the starting and ending time for that behaviour. A sample video was also used during the training session. No information about the purpose of the study was given to the coders.

### 3.1. Unwanted Behaviours

Two areas that were identified by the preliminary staff interviews and thus targeted during the video self-modeling training were* making unprovoked loud noises* and* invading personal space. *Thus, we first compared the occurrence of these behaviours on test videos taken before training to those taken after training.

In order to assess the change in* making unprovoked loud noises*, we compared the number of times coders recorded an unprovoked loud noise in “before” and “after” videos, averaged across coders. There was high interrater agreement in the frequency of* making unprovoked loud noises* (Kendall's tau-b = .83). There were more unprovoked loud noises recorded on videos taken before the intervention (M = 3.85, *sd*⁡ = 4.95) compared to videos taken after the intervention (M =,43, *sd*⁡ = .79; *t*(5) = 2.37, *P* = .03).

In order to assess the change in* invading personal space *while taking into account the varying lengths of the videos, we compared the proportion of time that JD was recorded invading personal space (time invading space divided by the total duration of the video) in the videos taken before the intervention compared to videos taken after the intervention, averaged across coders. There was high interrater agreement in the proportion of time spent of* invading personal space* (Kendall's tau-b = .83). JD spent a greater proportion of time invading personal space before intervention (M = .07, *sd*⁡ = .04) compared to after the intervention (M = .01, *sd*⁡ = .02; *t*(5) = 4.35, *P* = .004).

### 3.2. Desired Behaviours

There was no change in the number of times JD* initiated a handshake* before and after intervention. Before and after, the number of initiated handshakes, summed across the six videos and averaged across the raters, was 1.5.

There was no change in the number of times JD was seen* greeting politely* by the coders before and after intervention. Before the intervention, the number of times JD was coded as* greeting politely*, summed across the six videos and averaged across the raters, was 0.5, compared to 1.0 after.

In order to assess the change in* making eye contact *while taking into account the varying lengths of the videos, we compared the proportion of time that JD was recorded* making eye contact* (time making eye contact divided by the total duration of the video) in the videos taken before the intervention compared to videos taken after the intervention, averaged across coders. There was no significant difference. Proportion of time summed across 6 videos and averaged across coders before the intervention was .09, compared to .02 after intervention.

There was no change in the number of times JD was seen* responding to staff's requests* by the coders before and after intervention. Before the intervention, the number of times JD was coded as* responding to staff's requests*, summed across the six videos and averaged across the raters, was 8, compared to 6.5 after.

There was no change in the number of times JD was seen* responding to staff's questions *by the coders before and after intervention. Before the intervention, the number of times JD was coded as* responding to staff's questions*, summed across the six videos and averaged across the raters, was 10.5, compared to 5.5 after.

### 3.3. Staff Interviews

A second measure of interest was the change in problematic and appropriate behaviours described by staff in a reports gathered before and after the intervention. Five staff members of Salvation Army Lawson Ministries completed the survey before and after the intervention. One other staff member completed the survey before intervention but was no longer working with the participant after the intervention; her data were excluded from analyses.

In the survey conducted after intervention, when asked if there had been any improvement, staff reported improvements in several areas. Three of the five reported improvement with respect to invading personal space. Three of the five reported that JD was easier to redirect. Four of the five reported improvement with the greeting and handshake, or eye contact, including one staff member who reported that JD recognized the greeting from the video and was more likely to follow through with appropriate behaviours. Each of the five staff members interviewed reported some improvements in JD's behaviour following the video self-modeling intervention.

## 4. Discussion

This study revealed evidence that video self-modeling (VSM) can be an effective intervention with an adult with ASD who had previously shown persistent unwanted behaviours. Video coders who were blind to the hypothesis and did not know whether a given video was taken before or after intervention reported more instances of* making unprovoked loud noises* before the intervention compared to after and more time spent* invading personal space* before the intervention than after. This was consistent with staff interviews conducted after the intervention, which revealed that staff perceived an improvement with respect to invading personal space, ease of redirecting the participant, and verbal greetings, eye contact, and hand-shakes in the context of encountering someone.

Video self-modeling is an intervention in which the person who exhibits problematic behaviour is shown, on a recurrent schedule, a video showing himself or herself displaying only the target behaviour. The video explicitly describes and draws attention to this target behaviour. In the case of the current study, a video of the participant, JD, initiating a handshake, greeting politely, making eye contact, and respecting others' personal space was presented to him three times a week for four weeks.

Some of the behaviours of interest did not change significantly over the course of the intervention, according to our objective measures. For example, there was no statistically significant change in coder's recordings of* making eye contact, responding to staff's requests*, or* responding to staff's questions*. However, in a more subjective measure, staff members who were asked after the intervention to describe any differences in behaviour reported that JD was easier to redirect and was more likely to make eye contact during greetings. This contrast in results could be a result of the fact that the quantitative analyses did not have enough power to reveal these differences, or it could be results of an artificial report of behavioural improvements resulting from the demands inherent in asking this subjective question of staff members who were aware of the purpose of the intervention.

Although the intervention video targeted the prosocial interaction behaviours, the only statistically significant differences were in the negative behaviours. It is not clear why changes in negative, but not positive behaviours reached statistical significance. It could be that the reduction in negative behaviours resulted from his time being reallocated toward positive behaviours. In addition, it could be that the study design lacked sufficient power to reveal significant increases in positive behaviours.

Video self-modeling may be more effective than video modeling that portrays other people. Several reasons for this increased effectiveness have been proposed. The effectiveness of VSM may relate to the social deficit characteristics of those with autism. Video modeling might compensate for this deficit by minimizing social demand, empathy, and perspective taking [7]. In addition, Bandura suggested that the reason that VSM is so effective is related to its ability to improve the observer's self-efficacy, the observer's beliefs that he or she had the ability to perform a particular skill or would be successful at a specific endeavour [15]. It was also possible that VSM is effective to those who are primarily visual thinkers and learners, which may characterize some of those with autism [8].

Some limitations should be mentioned. Video self-modeling is a relatively time-consuming intervention. The current study employed a month-long intervention but might have failed to reveal the potential effectiveness of VSM. It is possible that a one-month intervention was not adequate for the participant to fully adopt the target behaviours depicted in the video. Additionally, evaluation of JD's behaviour was only based on six baseline videos taken before the intervention and six test videos taken after the intervention, potentially reducing the reliability of these findings. This relatively small data set might not have given a full representation of his behaviour. Finally, since this was a case study reporting results with a single individual, it does not shed light on the question of broader effectiveness within or beyond the autism community.

Finally, although improvements were seen in JD's negative behaviour, it is possible that this was due to the training he received in the course of creating the intervention video, rather than the recurrent viewing of the intervention videos. We do not think this is likely, since the duration of that training is minimal compared to the repeated viewing of the intervention video over several weeks, but future studies are needed to expand upon the current findings. If the intervention video were created more surreptitiously, catching desired behaviours that were displayed spontaneously, one could eliminate this confound.

This study suggests the effectiveness of VSM and is consistent with some other work that has shown that VSM is effective across a range of ages, behaviours, and abilities, in teaching a variety of skills, including math, life skills, social behaviours, and language, and when treating depression, stuttering, attention and behaviour disorders, and aggressive behaviours [8]. VSM may be incorporated into programs to help some adults with autism. Reducing challenging behaviours of individuals with autism could improve their social functioning and social relationships. These behavioural improvements might facilitate and enhance their involvement in the community. With increasing support on the usefulness of this intervention, video self-modeling has the potential to be used as a tool to effectively help some individuals with autism in the near future.

## Appendix

### Preintervention Survey

1. What are the challenging behaviours that Joel exhibits?
2. In what situation do you find this behaviour occurs?
3. Who does he usually show this behaviour with? (i.e., peers, staffs, strangers, parents)
4. What interventions have you tried when the behaviour occurs?
5. How often does this behaviour occur?
